# Electro-Medical Humbug

**Published:** 1875-02

**Authors:** 


An Electro-Medical Humbug.—There appeared, in the *Popular Science Monthly* of February, 1873, an article entitled "Is Electricity Life?" taken from the English *Belgravia Magazine.* Its admission to our pages was an editorial inadvertence, the article having been glanced at in haste, and only the first portion of it read. Its object, however, was to puff a quackish device of magnetic chains and bands, to be worn for the cure of nervous diseases. They were first called "Pulvermacher's Rings," and, having now been revived as "The Voltaic Armadillo," they are advertised as indorsed by the *Popular Science Monthly.* The advertiser says that the most eminent medical men of Europe and America approve their use, but none of their names are given, the sole authority quoted being the foreign writer in this magazine. Now, the publication of that article was a blunder; and the article itself is worthless and absurd; and if all editors, who happen to have been, at some time, the victims of careless oversight will copy this paragraph, they may help to protect a great number of stupid people with "rheumatics" and "neurology" against being humbugged.—*Popular Science Monthly.*

ORIENTAL PROGRESS.—In our last number we referred to some of the evidences of progress in Turkey. If *Galignani* is to be believed, the Sultan has taken another advance step in engaging Dr. Mary Walker and two of her pupils to attend to the health of his harem. "Some curiosity," says our Parisian cotemporary, "was caused on the boulevards on Friday by the appearance of three women attired in a singular costume, viz: large Zouave trousers, closed by gaiters, small gray paletots trimmed with black, and tall felt hats. On inquiry, they were found to be Miss Walker, an American medical practitioner, and two of her pupils, who were stopping at the Grand Hotel. The lady is about fifty years of age, and the apostle of the emancipation of women in the United States, and belongs to the sect of the Bloomerists. She is said to be on her way to Turkey, where she has just accepted the post of private physician of the Sultan's seraglio."

A SPECIMEN OF NOMENCLATURE.—Our chemical readers will doubtless be pleased to learn that a series of acids have been investigated by M. Hayduck. One is orthoamidotoluenesulphonic acid; and another diazorthoamidoparatoluenesulphonic acid. A knowledge of these is not indispensable to the practice of medicine. The action of tin and hydrochloric acid on nitrobromacetanilide gives rise to the hydrochloride of ethenylbromophenylenediamine.—*Medical and Surgical Reporter.*

